# Non-motor symptoms of Parkinson’s disease: the patient’s perspective

**DOI:** 10.1007/s00702-012-0928-2

**Published:** 2012-12-07

**Authors:** Kieran C. Breen, Gerda Drutyte

**Affiliations:** Parkinson’s UK, 215 Vauxhall Bridge Road, London, SW1V 1EJ UK

**Keywords:** Parkinson’s disease, Non-motor symptoms, Quality of life, Questionnaire

## Abstract

Parkinson’s disease (PD) can be manifested in many different ways. Although motor dysfunction represents the best characterised of the symptoms, the non-motor symptoms (NMS) of the condition can be equally disabling for people. These have been highlighted as being an issue of particular importance by people with PD. A comprehensive postal survey of members of the charity Parkinson’s UK took place in 2008. This resulted in returns from 10,101 people with PD. The self-completed Non-Motor Questionnaire (NMSQuest) and quality of life scale (PDQ-8) were contained within the survey. The results showed that the percentage of people with PD experiencing NMS increased with the duration of the disease. However, people who had the younger onset form of the condition reported a greater impact of NMS, particularly in the areas of memory, depression and sleep function. There is an inverse correlation between NMS and (PDQ-8 scale). A significant number of people with PD reported that they experienced problems with olfaction, taste, nocturia and constipation prior to diagnosis and these may help to serve as a future biomarker for the condition. Although our understanding of PD-associated NMS has increased considerably in the recent past, there is still a general lack of awareness of the importance of NMS for people with PD. Further research is required to identify the best treatments that should be employed to address them.

## Introduction

Parkinson’s disease (PD) is the second most common neurodegenerative disease affecting approximately 127,000 people in the UK alone. This is expected to rise to 160,000 by 2020 (Parkinson’s UK 2012). The importance of non-motor symptoms (NMS) for people with PD has gained a greater awareness in recent years (Lyons and Pahwa 2011). Reports have suggested that these can have a greater influence on the patient’s quality of life than the motor symptoms of the condition (Hinnell et al. 2012). This awareness has been accompanied by an increasing trend towards a more patient-centred approach to the diagnosis and treatment of the disease rather than concentrating on the treatment of the motor complications alone. However, the changes that underlie the NMS still remain unclear and they are not treated effectively well with dopamine-based drug therapies.

The need to further understand the basis and clinical burden of NMS has led to the development of a number of measurement tools including the patient-completed NMS Questionnaire (NMSQuest) (Chaudhuri et al. 2006; Martinez-Martin et al. 2007).This comprises 30 questions grouped into nine domains. It has been used widely both as a research tool as well as forming the basis for discussion, when a patient consults with a physician. This should lead to a more accurate diagnosis and a better management of the PD symptoms.

The generation of tools such as NMSQuest reflects a general increase in the overall development of patient-based questionnaires as it has been accepted that a combination of subjective and objective clinical measures is required to get a clear overall picture of the impact of PD on individual patients (Martinez-Martin et al. 2011). When combined, these will provide a greater understanding of all aspects of the disease and result in a better personalised therapeutic approach (Lyons and Pahwa 2011).

## Methods

The study followed a cross-sectional design. A postal survey containing 141 questions was sent to members of the Parkinson’s UK charity in 2007. There were a total of 10,101 valid responses from people with Parkinson’s with a mean age of 71 ± 9 (range 17–100). 59 % of the participants were male and 49 % of the total respondents have been diagnosed with PD for more than 5 years. A control group (*n* = 3, 209), who had not been diagnosed with PD (primarily the spouses and close relatives of the participants with PD) also completed the survey. The data were collected and analysed by the authors who retain ownership of the data. A significant amount of analysis has been carried out, since the survey was conducted.

The information obtained about the patients with PD included their age at diagnosis and the subsequent duration of their condition (six answer options from less than 1 year to more than 10 years). A number of specific health-related questionnaires were included in the survey in addition to general demographic data, and questions about local care and service provision. These included the NMSQuest (Chaudhuri et al. 2006) and the PDQ-8 short scale to rate quality of life (Jenkinson et al. 1997). The participants were asked to identify the NMS that they had experienced both before and after formal diagnosis of PD. The NMSQuest consists of a list of 30 common NMS that can then be grouped into nine domains for qualitative assessment purposes. The data was expressed as the percentage of participants who express one or more NMS in a specific domain.

No ethical consent was required to carry out this study as the data were submitted anonymously and members of the Parkinson’s UK had given prior consent to receive correspondence from the charity.

## Results

**Fig. 1:**
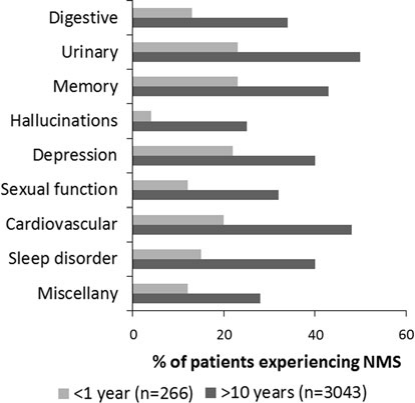
The percentage of survey participants, either diagnosed within 1 year prior to completing the survey or diagnosed more than 10 years previously who have experienced non-motor symptoms since their diagnosis

An average of 20 % of participants who had been diagnosed with PD within 1 year prior to completion of the questionnaire reported one or more NMS in at least one domain (Fig. 1). The primary domains highlighted among this group of patients included depression, memory and urinary problems. Hallucinations were reported by less than 5 % of the respondents. In contrast, there was a considerable increase in the percentage of NMS experienced by patients who had been diagnosed for more than 10 years. Again, hallucination was the lowest scoring domain and the most prevalent were urinary, memory and cardiovascular problems.

**Fig. 2:**
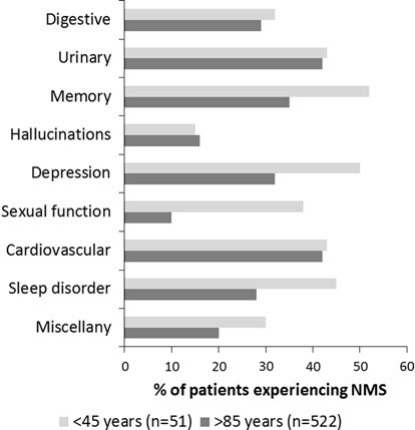
The percentage of survey participants, either under the age of 45 prior to completing the survey or over the age of 85, experiencing non-motor symptoms since their diagnosis

**Fig. 3:**
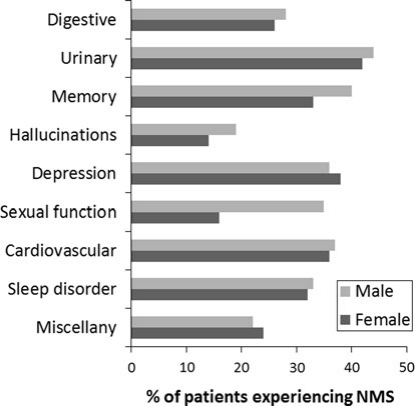
The percentage of survey participants experiencing non-motor symptoms stratified according to gender

When the groups were stratified according to age (less than 45 years and greater than 85 years), a different pattern was observed. A greater percentage of the younger people reported experiencing problems associated with sleep, sexual function, depression and memory when compared with the older age group (Fig. 2). There was very little inter-gender difference in the NMS profile apart from sexual function. 35 % of males expressed problems in this domain compared with 15 % of females (Fig. 3).

**Fig. 4:**
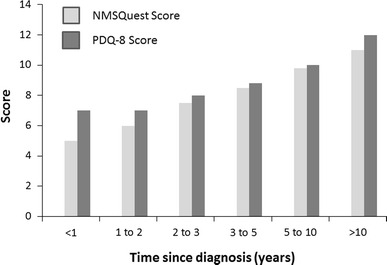
The NMSQuest score and PDQ-8 scores for groups with increasing disease duration. A higher score indicates an increased number of NMS and a worse quality of life

In order to measure the actual impact of NMS, the participants were asked to complete the PDQ-8 self-rating scale to assess their overall quality of life. The average number of NMS that people experienced increased accordingly to disease duration. This was associated with a parallel increase in the PDQ-8 score, indicating a correlation between the development of NMS and a worsening of the participants’ overall quality of life (Fig. 4).

**Fig. 5:**
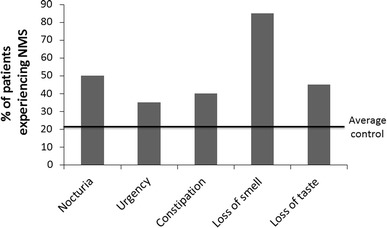
The percentage of survey participants reporting specific non-motor symptoms prior to their diagnosis with PD. The control line refers to the average percentage of a control (non-PD) population who indicated the non-motor symptoms that they experience. The line indicates the average percentage of the control population who currently experience the individual symptoms

Participants were asked to recall whether they experienced NMS prior to their diagnosis with PD and this was compared with a control group of people who answered the same questionnaire, but who had not been diagnosed with PD. These usually represented the spouses or close relatives of the participants with PD. Approximately 22 % of the control group reported experiencing at least one NMS in individual domains. However, there was a marked increase in the percentage of people with PD, who reported experiencing specific NMS prior to diagnosis. These specifically included nocturia, urgency, constipation, loss of smell and loss of taste (Fig. 5).

## Discussion

NMS are of particular importance for people with PD (Rahman et al. 2008) and it is vital to understand and appreciate the difficulties that they present in order to provide a more personalised therapy for those with the disease (Hinnell et al. 2012).

The percentage of people experiencing NMS in the current study was similar to that reported previously (Chaudhuri et al. 2006) and the spectrum across the individual domains was also in good agreement with published data (Martinez-Martin et al. 2007). The level of NMS has been reported to increase with disease duration (Barone et al. 2009) and the results of the current study confirmed this. However, it is interesting to identify the age-related changes in the individual domains of the NMSQuest in the patient sub-populations. Hallucinations are almost non-existent for the people who had been diagnosed with PD within 1 year of completing the survey (4 %), when compared with the overall levels of the other domains. However, this domain represented the largest-fold increase to 25 % in people who have had the disease for more than 10 years (Fig. 1). This may be associated with a general worsening of the condition and there are reports that people with more advanced PD are more likely to experience hallucinatory and other psychotic symptoms (Forsaa et al. 2010). However, this difference appears to be independent of age as there was no obvious difference between those under the age of 45 and those over 85 (Fig. 2). This suggests that disease duration and age could be considered as being independent variables.

In other domains, however, the percentage of respondents who reported experiencing NMS was higher in the younger participant cohort (Fig. 2). As those under 45 years would generally be expected to have had the disease for a shorter period of time than those over 85, the trend would be expected to be similar to the age-related differences (Fig. 1). However, the opposite was the case with a greater number of younger participants reporting having experienced NMS. This may represent a greater awareness of the NMS in the younger group and the impact that they would have on the person’s life and how this negatively influences their ability to carry out everyday activities. This correlates with a lower self-perceived quality of life as assessed by PDQ-8 score (data not shown). A previous report has proposed that people of a younger age predicted a worsening health status than older patients, i.e., the perception of the condition and its future progress was very different in the different age groups (Hinnell et al. 2012). The domains where there was a noticeable difference between the two age groups were sleep disorder, sexual function, depression and memory. However, problems in these areas have also been reported to be associated with general ageing as well as with the progression of PD (Krishnan et al. 2011). In the survey, the patients were specifically asked to identify those NMS that they had experienced since being diagnosed with PD. Therefore, older patients are unlikely to have experienced them only after being diagnosed with the condition so they may not associate them with the Parkinson’s but rather with general ageing. This would not be the case with younger people for whom the NMS would be considered as disease related. In addition, the diagnosis of early onset Parkinson’s will have a particular emotional impact on younger people. Depression is generally more common in people with PD, particularly following the initial diagnosis, where it is strongly influenced by the patients’ perceptions of their potential disabilities and the future progression of the disease (Schrag et al. 2000).There would also be an increased awareness of the implications of the disease on their future quality of life and this is likely to generate a significant level of anxiety. Younger people are also more likely to be sexually active, so any changes in their sexual abilities are more likely to be noted by the patients than in the older age group. The results also demonstrated that impaired sexual function has a considerable impact on males (Fig. 3).

There are increasing efforts to develop biomarkers of PD in order to obtain an earlier and more accurate diagnosis of the condition. A recent study reported that up to 20 % of people had been mis-diagnosed with PD and are therefore not being treated appropriately(Newman et al. 2009). In addition, future neuroprotective and neurorestoative therapies will need to be administered at an early pre-motor stage of the condition, if they are to be fully effective. In order to achieve this, reliable biomarkers must be developed. Patients have frequently reported prodromal symptoms in advance of the initial diagnosis of PD (Gaenslen et al. 2011). The participants in the present study reported the development of loss of smell and taste in addition to problems with nocturia, urgency and constipation as key pre-motor symptoms when compared with the prevalence of these symptoms in an age-matched control group. This is in agreement with previous studies which have specifically reported olfactory loss as an early symptom of Parkinson’s (Haehner et al. 2011). However, other more robust pre-motor symptoms such as REM sleep behavioural disorder and day-time sleepiness should be taken into account when developing a battery of premotor symptoms as a marker of early PD (Iranzo 2011). There must also be caution on the level of emphasis placed on retrospective questioning as this may be subject to an element of recall bias.

Patient self-rating scales are generating an increased level of interest and importance within the clinical setting. It is acknowledged that patients frequently know more about the symptoms and impact of their conditions than their physicians. Therefore, a combination of the objective medical rating scales, (e.g. UPDRS, Hoehn and Yahr) along with the more subjective patient-based measures such as health-related quality of life will provide a more accurate indication of a patient’s overall condition (Den Oudsten et al. 2007). An example of this is the phenomenon of “wearing-off” associated with fluctuating drug levels. Following the development of a patient “Wearing-Off Questionnaire”, it was noted that patients reported wearing-off more frequently (57.1 %) than was observed by a physician (29.4 %) during a routine clinical appointment (Stacey and Hauser 2007). A large number of such scales exist, some of which are generic, while others are specific for PD (Martinez-Martin et al. 2011). However, further research is required to assess the usefulness of these scales in a clinical setting and which ones provide the relevant information being sought by the physician.

The participants in this study were selected from membership of a UK charity. This could be considered to be biased, as the demographics of the group may not reflect exactly those of the general population. However, a large number of participants are likely to largely overcome any such bias. This is underlined by the fact that the percentage of people experiencing NMS as well as the general trend across the domains is in line with reports from the previous studies.

In conclusion, we have demonstrated a marked expression of NMS in a large population of people with PD. While the percentage expressing the symptoms increased with disease duration, this was independent of age. There were also considerable differences between the individual NMS domains expressed by specific patient sub-groups. This highlights the importance of specific NMS domains to individual patients and this should be taken into consideration when treatment is being considered.
